# Antarctic daily mesoscale air temperature dataset derived from MODIS land and ice surface temperature

**DOI:** 10.1038/s41597-023-02720-z

**Published:** 2023-11-27

**Authors:** Eva Bendix Nielsen, Marwan Katurji, Peyman Zawar-Reza, Hanna Meyer

**Affiliations:** 1 Centre for Atmospheric Research, School of Earth and Environment at University of Canterbury, Christchurch, New Zealand; 2 Institute of Landscape Ecology at University of Münster, Münster, Germany

**Keywords:** Climate change, Cryospheric science

## Abstract

Knowledge about local air temperature variations and extremes in Antarctica is of large interest to many polar disciplines such as climatology, glaciology, hydrology, and ecology and it is a key variable to understand climate change. Due to the remote and harsh conditions of Antarctica’s environment, the distribution of air temperature observations from Automatic Weather Stations is notably sparse across the region. Previous studies have shown that satellite-derived land and ice surface temperatures can be used as a suitable proxy for air temperature. Here, we developed a daily near-surface air temperature dataset, AntAir ICE for terrestrial Antarctica and the surrounding ice shelves by modelling air temperature from MODIS skin temperature for the period 2003 to 2021 using a linear model. AntAir ICE has a daily temporal resolution and a gridded spatial resolution of 1 km^2^. AntAir ICE has a higher accuracy in reproducing *in-situ* measured air temperature when compared with the well-established climate re-analysis model ERA5 and a higher spatial resolution which highlights its potential for monitoring temperature patterns in Antarctica.

## Background & Summary

Local near-surface air temperature variations and extreme temperatures in Antarctica resulting from mesoscale climate processes can have a large effect on the biodiversity^1–3^ and have a significant influence on hydrological^4^ and glaciological processes^5^. With a globally warming climate, Antarctica has been the focus of climate research for its impacted melting ice shelves, sea ice changes, and surface mass balance^6–9^. There has been a growing focus throughout several regions of Antarctica on localized climate extremes caused by foehn wind warming events^10–12^ impacting the ice shelf mass balance^13^ and thereby being an important component in the breakup of ice shelves^14^. Hydrological extremes in areas of high biodiversity have also been linked to foehn warming events^15^. Shorter term climate perturbations and extreme meteorological events have an important indirect influence on species distribution and ecosystem functioning through its contribution to temporal availability of meltwater^16,17^. The availability of a high-resolution air temperature dataset over multiple decades is therefore important to analyse spatio-temporal variability and understand the effects of these mesoscale temperature variations and trends on the physical and biological systems across entire Antarctica.

Hourly *in-situ* measurements of air temperature are available from Automatic Weather Stations (AWS), but with a low spatial density due to cost and logistical issues in operating in such a remote location. Therefore, AWS data are not sufficient for a comprehensive spatio-temporal analysis of climate variables. Atmospheric reanalysis products such as ERA5 also provide Antarctic-wide near-surface temperatures with a high temporal resolution, but with a grid spacing of 31 km, which is not sufficient for resolving local patterns and processes causing near-surface temperature variability. Numerical weather prediction models such as The Antarctic Mesoscale Prediction System (AMPS), provide both a high temporal and spatial resolution air temperature with up to an hourly temporal and approximately 1 km spatial resolution^18^, but solely for selected regions such as the Antarctic Peninsula and the Ross Sea Region and currently only available for a limited span of years.

Remote sensing data in the thermal bands can be used as proxies for air temperature^19^, and may therefore be a solution to this demand for high spatial-temporal resolution products capable of estimating mesoscale dynamics. The MODerate-resolution Imaging Spectroradiometer (MODIS) is onboard the Aqua and Terra satellites and with their polar orbits, they provide Antarctic wide imagery of a large range of products several times a day with 1 km spatial grid resolution^20^. Studies from all over the world including the polar regions have shown that the MODIS land surface temperature (LST) and ice surface temperature (IST) products are good proxies for air temperatures^21–32^. As *in-situ* skin temperature measurements are sparse for Antarctica, AWS measurements of air temperature have in previously studies been used for validating the MODIS skin temperature products. Wang *et al*.^27^ compared AWS measurements with MODIS LST over the Lambert Glacier Basin in East Antarctica and proposed that there is great potential in using MODIS LST for improving the reconstruction of spatio-temporal variability of temperature in Antarctica. A study of the MODIS IST by Shuman *et al*.^33^ concluded that the MODIS IST product has the consistency to provide knowledge of the surface temperature of the Greenland Ice sheet. They furthermore suggested an empirical correction to refine the correlation between air temperature measured at 2 m height by AWS and MODIS IST values due to a cold bias between the MODIS LST and IST and air temperature measured in AWS at 1 m to 3 m height in arctic regions^29,33,34^. This apparent cold bias is due to a near-surface temperature inversion with thermal stratification near the snow surface^29^. Using AWS measurements as ground truth for creating a validated air temperature dataset from MODIS surface temperature products was published by Hooker *et al*.^30^ but Antarctica was excluded from their global dataset. Recently Zhang *et al*.^31^ developed an Antarctic Near-Surface Air Temperature based on MODIS observations and AWS measurements but in monthly means and a 5.6 km spatial grid resolution which is not enough to resolve these mesoscale warming phenomena. The previous case studies in Antarctica are also only based on either MODIS LST or MODIS IST which does not provide comprehensive temperature information for identifying warming that extend along the terrestrial costal margin.

Meyer *et al*.^28^ showed the utility of training machine learning models and a linear regression technique to make predictions of the near-surface air temperature over Antarctica based on MODIS LST values for 2013. The models were trained on air temperature measured at 3 m height at the exact time of a satellite overpass. It was concluded that the methods were promising but further research needed to include more training samples from a longer period to improve the models. The study by Meyer *et al*.^28^ further showed that a linear model was comparable to the more advanced machine learning algorithms. Tree-based machine learning models have large issues with extrapolation outside the range of the original training dataset^35,36^. With a shift in the distribution of measured temperatures in Antarctica in the past decades and an increasing number of extreme high temperatures^37^ a model must be robust towards these extremes and capable of extrapolation. This would favour the applicability of a linear model as simpler but yet robust model for this purpose, over machine learning models.

Here, we develop AntAir ICE, a near-surface air temperature dataset for the entire terrestrial Antarctica, the ice shelves, and the seasonal sea ice around Antarctica by using MODIS IST and LST as a proxy for near-surface temperature. This study uses air temperature measured by 117 AWSs at 3 m height for ground truth for this calibration. The linear relation between the daily mean of MODIS IST and LST and daily mean air temperature measured at 3 m height was established and then applied on the full MODIS record for the past 19 years. The developed AntAir ICE dataset has a spatial grid resolution of 1 km and represents daily average air temperature. The dataset is developed for the purpose of researching local temperature variations and extremes that are currently not well represented in available numerical climate models and reanalysis products.

## Methods

**Fig. 1:**
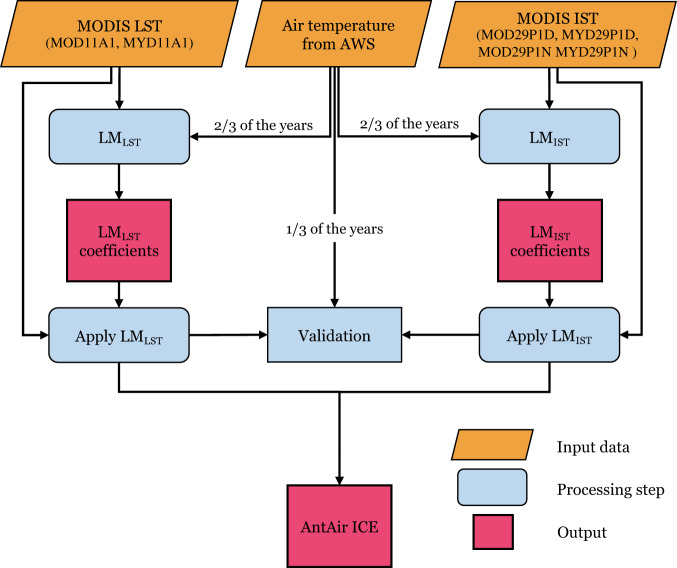
Schematic overview of the processing steps to generate AntAir ICE. The output of this process was the coefficients for the linear relationship between the MODIS LST and IST and air temperature measured in AWS separately. These linear models (LM_LST_ and LM_IST_) were then applied to the full MODIS record creating the AntAir ICE near-surface air temperature dataset.

The flowchart (Fig. 1) shows the schematic overview of the processing steps to generate the near-surface air temperature dataset, AntAir ICE.

### Remote sensing data

The MODIS sensor delivers the land surface temperature and ice surface temperature as a standard product also referred to as skin temperature or surface radiometric temperature^38^. The MODIS IST product is a sea ice product available for ice shelves and surrounding sea ice and the MODIS LST product is available for continental Antarctica. The MODIS skin temperature is estimated using a generalized split-window algorithm from the emissivity from MODIS sensor radiance data product band 31 and 32^39^. The emissivity for the LST product is estimated based on information about the land cover type, the lower-boundary air surface temperature and atmospheric column water vapour^39^, whereas the IST algorithm uses a fixed snow/ice emissivity^40^. In the presence of clouds, the MODIS cloud mask product, MOD35 and MYD35, is used for removing cloud contaminated pixels in both the MODIS LST and IST product. For MODIS IST a pixel is considered cloud free if the cloud mask indicates that there is a 66% chance or higher that the pixel is clear and this probability is increased to 95% or higher for the MODIS LST products^40^. For the MODIS all pixels are furthermore quality controlled and the quality information for LST is saved in the products quality flag. Besides a swath for each satellite overpass, the MODIS LST and MODIS IST products are compiled into a night and daytime product by selecting the best quality measurements for that day. The MODIS LST product is divided into night and daytime products based on local solar time^40^ and the products over Antarctica are therefore not reflecting the solar position but a time interval, whereas the MODIS IST products are divided into a day and night product based on the solar zenith angle, and 0° to 85° is defined as daytime^40^.

In this study, the MOD11A1 and MYD11A1 LST products^41^ containing both a daily daytime and nighttime skin temperature product each at a 1 km grid resolution, were used. The data is retrieved from the NASA LPDAAC website (https://lpdaac.usgs.gov/) part of collection 6 and automatically downloaded using the R package RGISTools^42^. For IST the day products^40^ MOD29P1D, MYD29P1D, and night products MOD29P1N and MYD29P1N that is available daily and in a 1 km grid resolution from collection 6 was used. The data was accessed through NASA NSIDC DAAC website (https://nsidc.org/) and downloaded using the available python script from the NSIDC DAAC Data Access Tool. It is a well-known issue that the MODIS LST product still contains pixels with clouds that the cloud mask product cannot detect^43,44^. In this case, LST or IST values reflect cloud top temperatures which are usually far below the skin temperature measured on surrounding days. A threshold of two times the standard deviation below the mean was applied for each pixel to remove this cloud contamination as also used in previous studies of MODIS skin temperature products^44^. Further preprocessing was applied to the MODIS IST since a snow or ice surface with a skin temperature above 0 °C is not possible in the MODIS IST product^45^, but the MODIS IST is known to have these wrongly estimated pixels^44,45^. Following the procedure from Yu *et al*.^44^, all pixels with a MODIS IST above 0 °C were removed.

### Automatic weather station data

**Fig. 2:**
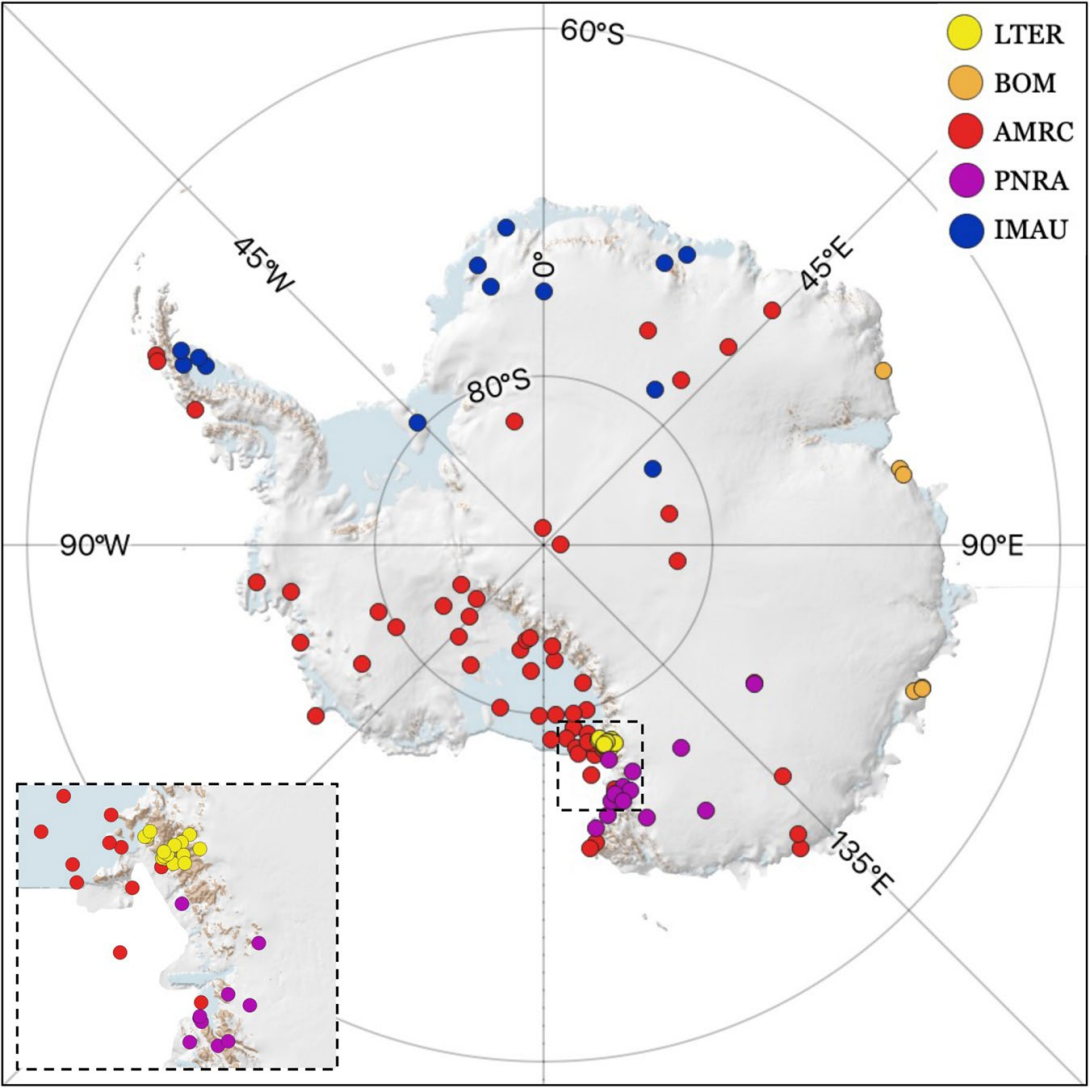
Automatic weather stations, AWS used in this study. Colors indicate source of data: The Long-Term Ecological Research program (LTER), the United States Department of Agriculture or the Antarctic Meteorological Research Center at the University of Wisconsin (AMRC), Institute for Marine and Atmospheric Research, Utrecht University (IMAU), Meteo-Climatological Observatory of Italy (PNRA), and Antarctic Climate Data Collected by Australian Agencies (BOM). Basemap from Quantarctica^56^.

Measurements of daily mean air temperature from AWS (T_air_) were used as the ground truth in this study for modelling and validation. The spatial distribution of the available AWS from five different providers used for AntAir ICE throughout Antarctica are illustrated in Fig. 2. Data from AWS were obtained from Meteo-Climatological Observatory of Italy^46^, the Long-Term Ecological Research program^47^, the United States Department of Agriculture or the Antarctic Meteorological Research Center at the University of Wisconsin^48^, Institute for Marine and Atmospheric Research, Utrecht University^49^ and Antarctic Climate Data Collected by Australian Agencies^50^. There is a total of 117 stations (compared to 70 stations used for AntAir version 1 by Meyer *et al*.^51^) available in this study. All stations provided 15-minute to hourly sampling intervals of air temperature in 2 or 3 m height and only station records with the complete 24-hour period of measurements aggregated into daily averages. The temperature sensors on the stations have a shield for natural ventilation that prevents heating from direct sunlight and the uncertainty lies within ± 0.5 °C^52^.

### Compilation of training and validation data

For each AWS location a 19-year record of MODIS skin temperature was derived by extracting the MODIS time series of the grid cell containing the station. The daily mean of either MODIS LST or IST were matched with the T_air_ for the corresponding station depending on the location of the station. For LST only days with 4 available scenes with good quality were selected based on the pixel’s qualify flag. There were in total 47,865 cloud free data points of matching daily MODIS LST and measured T_air_ within the period from 2003 to 2021. For the IST the day and night products are based on the solar zenith angle and due to the high latitude, there will only be a day (or night) product available during the austral summer (austral winter). However, due to the extent of Antarctica, this is only valid for the most southern part of Antarctica. A latitude threshold of 75°S was therefore chosen. For 75°S latitude and northwards there must be either a day and night product together or all 4 scenes available. For 75°S to 90°S latitude one scene during the summer period and at least two scenes during the winter period were selected. There were in total 38,809 cloud free data points of matching daily MODIS IST and measured T_air_ within the period from 2003 up to and including 2021. Every third year was used for model validation (2003, 2006, 2009, 2012, 2015, 2018, 2021), and the rest for fitting the model.

### Model training and validation

**Fig. 3:**
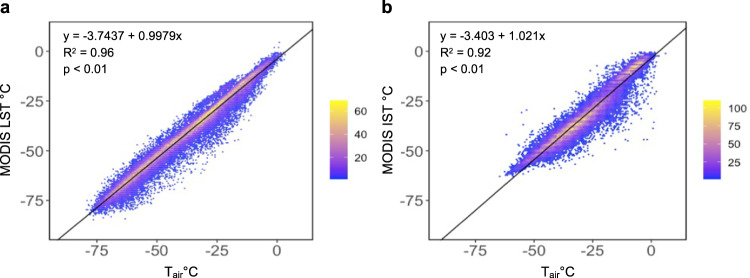
Linear regression line in black between daily mean air temperature measured in Automatic Weather Stations and daily mean MODIS skin temperature. (**a**) MODIS LST and (**b**) MODIS IST.

A linear regression model between the daily skin temperature from MODIS and T_air_ was derived using the MODIS data as predictor and the corresponding air temperature as measured by the stations as the response. Due to the difference in the MODIS LST and IST products’ temperature range and environment, two separate models were made for each product referred to as LM_LST_ and LM_IST_. The linear relation between the measured T_air_ and daily mean MODIS LST and MODIS IST are shown in Fig. 3a,b respectively (p-value < 0.01).

The trained models were then used to predict the held-back validation data for the purpose of model testing. The models were used to make predictions for the entire spatial time series for each product and then spatially merged into one product. The final predictions are the continues near-surface air temperature dataset in a daily temporal resolution and 1 km grid resolution for the period 2003–2021, AntAir ICE. The daily mean near-surface air temperature from the LM_LST_ and the LM_IST_ model will be denoted T_AntAir_ in the following.

## Data Records

The AntAir ICE dataset is published on PANGEA^53^. The geographic coverage of the AntAir ICE dataset includes the entire continent of Antarctica, the surrounding ice shelves, and partly sea ice in 1 km spatial resolution and a daily average temporal resolution for the period 2003–2021, inclusive. The dataset is in GeoTIFF format and in the Antarctic Polar Stereographic projection (EPSG 3031) with one file per day. Each day is a spatial raster group with two layers; the first layer is the predicted air temperature for that day in degree Celsius using a scaling factor of 0.1, the second layer is the number of available MODIS scenes for each grid cell for that day, ranging from 0 to 4. Areas with cloud contamination or without sea ice are marked with no data. Files are named AntAir_ICE_<YYYY>_<DOY>.tif, where <YYYY> represents the year and <DOY> represents the day of the year. Files are divided into quarters with January, February, March as 1, April, May, June as 2, July, August, September as 3 and October, November, and December as 4, for each year (2003–2021) and compressed to a ZIP files. Data are also available on New Zealand modelling consortium open environmental digital library (https://www.envlib.org) with access through the tethysts python package.

## Technical Validation

**Fig. 4:**
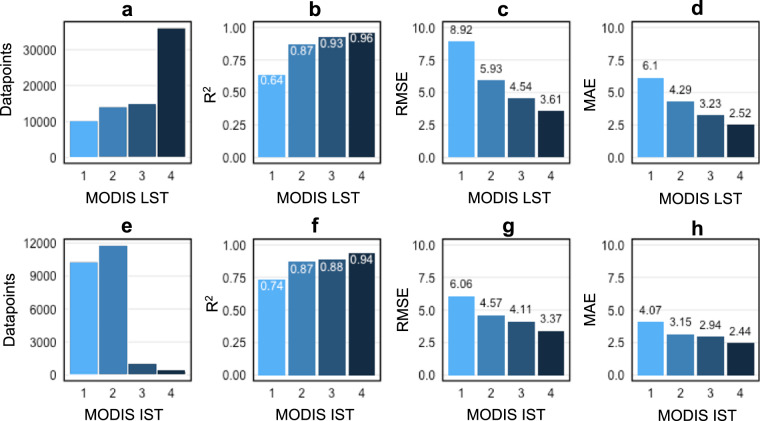
The relationship between the number of cloud free pixels, and the RMSE, MAE, and R^2^. (**a**–**d**) from the linear model for LST and (**e**–**h**) from the linear model for IST.

Temporal validation of the LM_LST_ and LM_IST_ models was done based on every third year (2003, 2006, 2009, 2012, 2015, 2018, 2021) of measurements from all AWS and on days with no clouds and good quality according to the flag. As performance measures, the Mean Absolute Error (MAE), Root Mean Square Error (RMSE), R-squared (R^2^) and Mean Bias (MB) were used (Table 1). The LM_LST_ model has a MAE of 2.26 °C and a R^2^ value of 0.97 which indicates that the model could well estimate the overall patterns in air temperature. The LM_IST_ model does have a slightly lower, but still very high R^2^ value of 0.93 and a MAE of 2.66 °C. Since the final AntAir ICE temperature dataset is containing days with cloud contaminated scenes and lower quality pixels, the RMSE, MAE, R-squared and MB were additionally calculated for the validation years for the full non-filtered days (Table 1). The models performance slightly decreased for both, the LM_LST_ model (MAE = 3.48 °C, R^2^ = 0.92) and LM_IST_ model (MAE = 3.52 °C, and R^2^ = 0.82) for the entire dataset but the models were still capable of predicting air temperature within reasonable error. The relationship between the missing MODIS scenes and the performance of the models is illustrated in Fig. 4 where it is clear that the LM_IST_ model is not as sensitive to requiring all 4 scenes as the LM_LST_ model. The daily availability of 4 cloud free scenes in the MODIS LST is also more occurrent (Fig. 4a) than for the MODIS IST (Fig. 4e). For the LM_LST_ model, the R-squared was low for pixels with only one available scene (R^2^ = 0.64) but it increases drastically when two scenes were available (R^2^ = 0.87). As the final datasets contain information in the metadata about the number of available scenes the end user can choose to not include the cloud contaminated days. The continued validation and comparison were based on the temporal validation set with four available scenes and good quality flags.

**Table 1:** Temporal validation of the LM_LST_ and LM_IST_ using Mean Absolute Error (MAE), Root Mean Square Error (RMSE), R-squared (R^2^) and Mean Bias (MB).

	LM_LST_				LM_IST_				AntAir ICE			
	MAE	RMSE	R^2^	MB	MAE	RMSE	R^2^	MB	MAE	RMSE	R^2^	MB
Cloud free	2.26	3.30	0.97	−0.66	2.66	3.63	0.93	−0.09	2.73	3.94	0.95	−0.61
DJF	1.63	2.16	0.96	0.02	2.25	3.31	0.89	−0.06	3.03	4.34	0.93	−1.02
MAM	2.71	3.79	0.94	−0.12	2.89	3.99	0.82	−0.41	3.06	4.28	0.93	−0.68
JJA	2.86	4.18	0.93	−0.77	2.72	3.54	0.83	0.29	3.37	4.91	0.91	−1.51
SON	2.11	3.14	0.96	−0.84	2.62	3.46	0.93	−0.08	2.66	3.81	0.94	−0.99
Full dataset	3.48	5.27	0.92	−2.48	3.52	5.21	0.82	−1.25	3.80	5.91	0.88	−2.06

Temporal validation is based on the test dataset using the same filtering as the training dataset. The full dataset is the temporal validation if any number of overpasses is used. DJF is for December-February, MAM is for Marts to May, JJA is for June to August, SON is for September to November for the test dataset using the same filtering as the training dataset. AntAir ICE was validated against available AWS records from the AntAWS dataset.

The newly published Antarctic AWS dataset AntAWS^52^ contains quality controlled daily temperature records from 216 AWS records from the period 1980–2021. For validation AntAir ICE, temperatures for the temporal validation period were compared to the 176 available AWS records from AntAWS using performance measures MAE, RMSE, R^2^ and MB (Table 1). The R-squared values were still high compared to the two models individually and similarly the MAE for cloud free days (MAE = 2.73 °C) was only slightly increased when the quality controlled AntAWS dataset is used for validation.

### Seasonal variation

**Fig. 5:**
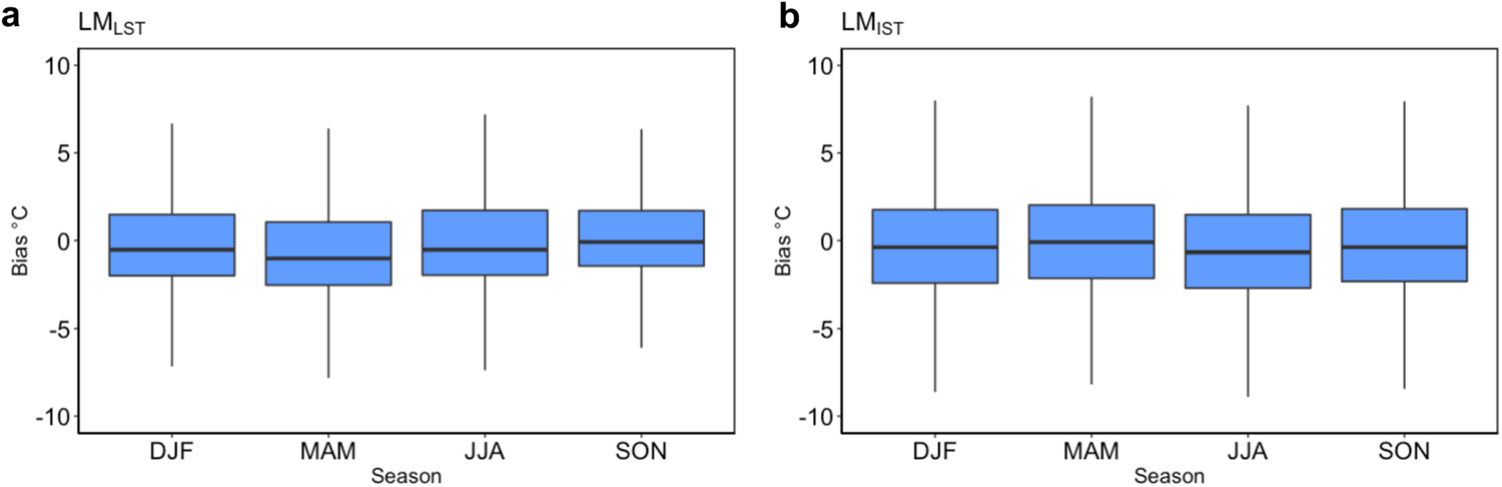
Seasonal bias between model and *in-situ* air temperature measurements. Bias in °C is the difference between the measured air temperature from automatic weather stations and the models’ prediction. (**a**) is for the LM_LST_ and (**b**) is for the LM_IST_. The Bias is measured for each seasons December, January, and February, (DJF), September, October, November (SON), March, April, and May (MAM) and June, July, and August (JJA) for all weather stations and for the temporal validation years.

Due to the location of Antarctica, there is a large seasonal variation driven by the change in incoming solar radiation and it is therefore important to test the LM_LST_ and the LM_IST_ models’ performance for each season in the validation years (Table 1). There was a clear trend that the summer months, December, January, and February, (DJF) have the lowest RMSE and MAE for both LM_LST_ and LM_IST_ models. The spring season, September, October, and November (SON) also showed a low RMSE and MAE for both models. The autumn, March, April, and May (MAM) and winter months, June, July, and August, (JJA) had the highest RMSE and MAE for both models. This larger bias is expected due to the highly variable temperature conditions in winter caused by wind, which is only partly reflected in AntAir ICE, however, the MAE did not exceed 3 °C or any season for either the LM_LST_ or LM_IST_ models. The bias, calculated as the difference between the T_air_ from the AWS measurements and the T_AntAir_, for each season for the temporal validation years is illustrated in Fig. 5_._ The box plot indicates that for the majority of data, the bias was close to zero.

### Trend comparison

**Fig. 6:**
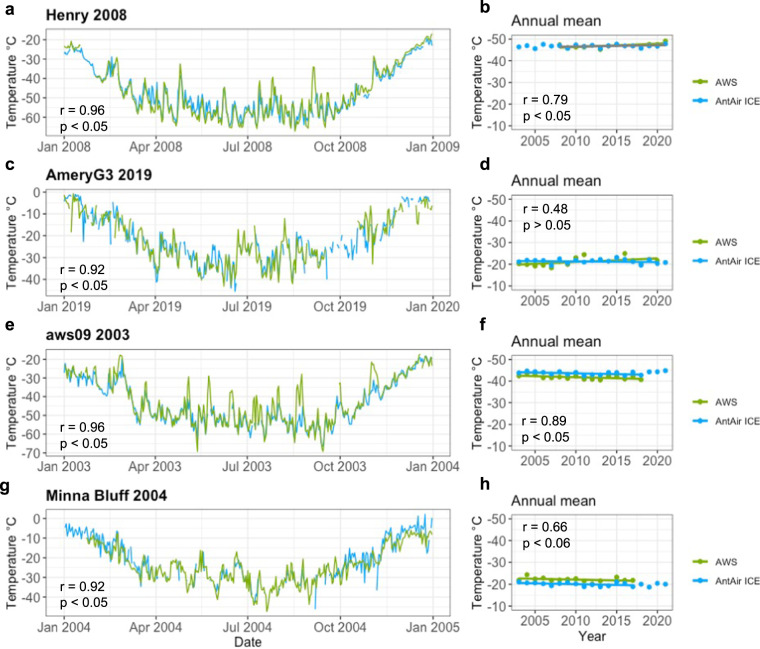
Time series of daily mean air temperature from AntAir ICE in blue and AntAWS data in green for AWS (**a**) Henry 2008, (**c**) AmeryG3 2019, (**e**) AWS09 2003 and (**g**) Minna Bluff 2004. Mean annual temperature from AntAir ICE in blue and AntAWS data in green for AWS (**b**) Henry, (**d**) AmeryG3, (**f**) AWS09 and (**h**) Minna Bluff with temporal trend line for years with available AWS record.

To analyse how the AntAir ICE is comparing to T_air_ time series and trend, the records of daily mean air temperature in the AWS Henry, AmeryG3, AWS09 and Minna Bluff were compared using the AWS data from AntAWS^52^ along with the temporal trend in annual mean for both the station and correlating AntAir ICE grid cell (Fig. 6). The annual mean from AntAir ICE also includes cloud interfered days where not all four scenes were available, nevertheless the correlation between the T_air_ from the AWS measurements and the T_AntAir_ is significant for all four stations throughout the year. The annual mean trends were furthermore very similar and significant for all stations except AmeryG3. At AmeryG3 and Minna bluff there were cold spikes that could be related to cloud contamination.

### Comparison with ERA5 reanalysis

ERA5^54^ is a widely used climate reanalysis dataset for air temperature, however, with a grid spacing of 31 km, it is in some cases not capable of resolving local processes causing near-surface temperature variability. By comparing AntAir ICE to ERA5 performance in multiple Antarctic regions this study can illustrate how the high resolution of AntAir ICE is improving the understanding of spatial temperature patterns. The ERA5 temperature data were obtained from ECMWF (https:// apps.ecmwf.int/datasets/) from 2003 to 2021. Using a similar method as Zhu *et al*.^55^ the hourly 2 m temperature record from the ERA5 was extracted from the nearest grid locations of all 117 AWS and a daily mean was produced, T_ERA5_. Every third year was used for model validation (2003, 2006, 2009, 2012, 2015, 2018) were selected for comparing ERA5 to the *in-situ* measured T_air_, which excluding 2021 the same years used for validation of AntAir ICE. To account for the elevation difference between the selected ERA5 grid cell and AWS location, a correction in elevation using the dry adiabatic lapse rate of 9.8 °C/km was applied for all 117 stations, and the MAE, RMSE, and R^2^ from the comparison was calculated (Table 2). The lapse rate correction improves ERA5 performance significantly, with a RMSE = 4.31 °C, MAE = 3.02 °C, and R^2^ = 0.92, which were a higher value for RMSE and MAE and a lover R-squared than the LM_LST_ and LM_IST_ models’ temporal validation.

**Table 2:** Mean Absolute Error (MAE), Root Mean Square Error (RMSE), and R-squared (R^2^) for temporal validation of ERA5 for all 117 Automatic Weather Stations.

	ERA 5		
	MAE	RMSE	R^2^
Temporal validation	3.47	4.47	0.91
Temporal validation - lapse rate correction	3.02	4.31	0.92
DJF	3.38	4.73	0.92
MAM	3.51	4.85	0.88
JJA	3.97	5.42	0.86
SON	2.63	3.74	0.93

ERA 5 air temperature measurement was also corrected for the elevation difference between AWS and grid height with a dry adiabatic lapse rate correction and RMSE, MAE and R^2^ were calculated again. DJF is for December-February, MAM is for Marts to May, JJA is for June to August, SON is for September to November for the elevation corrected ERA5 data.

**Fig. 7:**
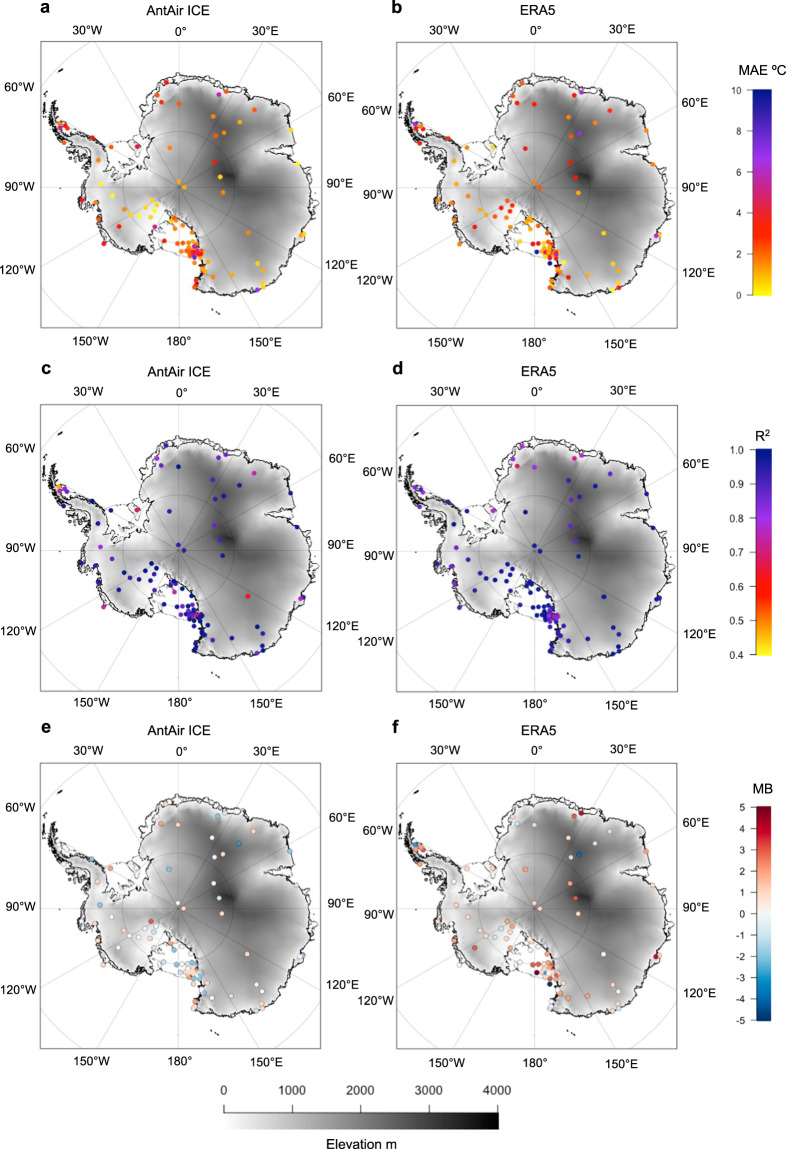
Prediction error in the estimation of air temperature. Mean absolute Error, MAE for all 117 automatic weather stations for (**a**) AntAir ICE and (**b**) ERA5 for the temporal validation using dry lapse rate correction. R-squared for c) AntAir ICE and d) ERA5. Mean Bias for e) AntAir ICE and f) ERA5. Plotted on top of a terrain model from the Reference Elevation Model of Antarctica^57^.

The spatial variations in AntAir ICE and ERA5 performance measured as MAE when compared to *in-situ* measured T_air_ (Fig. 7a,b) indicated a very similar MAE for the two temperature datasets. For T_AntAir_ 70% of the AWS locations have a MAE below 3 °C whereas it was 67.5% for ERA5. The AWS located in West Antarctica seemed to have a lower MAE for T_AntAir_ than for T_ERA5_. The Ross Sea Region around Ross Island have for T_ERA5_ two AWS locations with very high MAE, which could be due to the very complex terrain that ERA5 was not as capable of resolving. The spatial variations in AntAir ICE and ERA5 performance measured as R^2^ were very similar for the two datasets with most stations above R^2^ = 0.8 (Fig. 7c,d). For the stations located in West Antarctica and the Antarctica Peninsula the mean bias were lower for AntAir ICE than for ERA5 (Fig. 7d,e). The East Antarctic Plateau stations also had a low mean bias in AntAir ICE (Fig. 7e) showing a good performance.

**Fig. 8:**
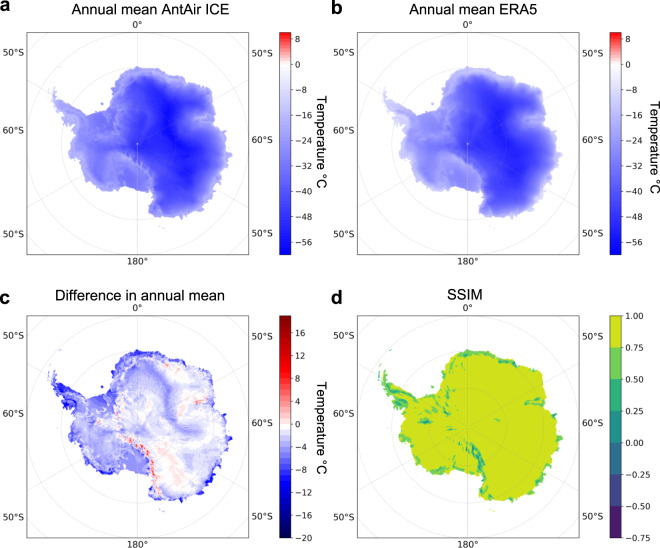
(**a**) Annual mean temperature from AntAir ICE from 2003–2021 resampled to ERA5 grid resolution. (**b**) Annual mean temperature from ERA5 from 2003–2021. (**c**) difference between AntAir ICE and ERA5 annual mean temperature and (**d**) structural similarity index between AntAir ICE and ERA5 annual mean temperature.

To compare the similarity spatially between ERA5 and AntAir ICE the annual mean from 2003 to 2021 was calculated for both datasets and AntAir ICE was resampled to ERA5 resolution (Fig. 8a,b). The difference between the annual mean in Fig. 8c shows a very similar pattern but there is a clear higher warming pattern in AntAir ICE along the Transantarctic Mountains and a colder bias on the Antarctic Peninsula. To investigate the structural similarity in the two annual means a structural similarity index (SSIM) was used. SSIM can be used as a tool to assess perceived changes in structural information between two spatial inputs and the metric has been found to be a more meaningful assessment of structural changes compared to a traditional metrics like Mean Square Error (MSE). A SSIM of 1 represents a perfect match and it was found that the mean SSIM between AntAir ICE and ERA5 annual mean was SSIM = 0.74. There was a clear difference in the structural similarity index (Fig. 8d) and difference between ERA5 and AntAir ICE in areas with complex terrain. Especially around the Transantarctic Mountains there was a warmer trend in AntAir ICE which could be due to this area is exposed to warming katabatic outflows and these warming patterns were not resolved in ERA5. This was also supported by the lower MAE and MB in AntAir ICE than ERA5 for the stations in this area (Fig. 7).

**Fig. 9:**
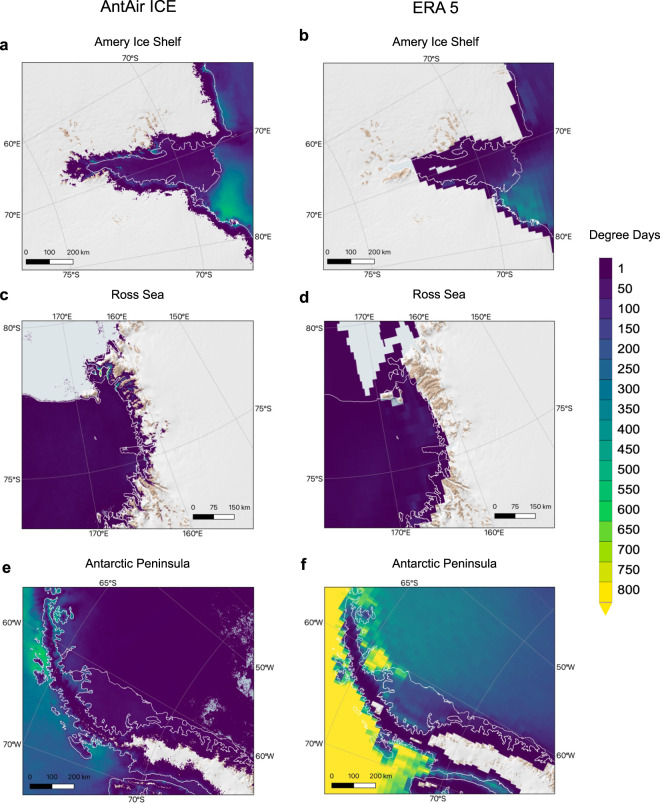
Accumulative days with above 0 °C air temperature for summer season DJF 2003–2021. Data from AntAir ICE for (**a**) Amery Ice Shelf, (**c**) Ross Sea and (**e**) Antarctic Peninsula. Data from ERA5 for (**b**) Amery Ice Shelf, (**d**) Ross Sea and (**f**) Antarctic Peninsula. Basemap from Quantarctica^56^.

To illustrate the uniqueness of this high-resolution near-surface air temperature dataset in comparison to the ERA5, the accumulative sum of days with temperature above 0 °C for each pixel in three different regions of Antarctica for the full 19-year temperature record for DJF were calculated from ERA5 and AntAir ICE respectively (Fig. 9). It is clear to see how AntAir ICE resolves inter-valley variability in the McMurdo Dry valleys in the Ross Sea Region (Fig. 9c) compared to ERA5 (Fig. 9d). The sea ice areas in the Antarctic Peninsula seemed to have a higher occurrence of above 0 °C for ERA5 (Fig. 9f) than for AntAir ICE (Fig. 9e). This is caused by the fact that AntAir ICE were only available for that area when sea ice was present due to the nature of the MODIS IST product whereas ERA5 provides air temperature for the full period independently of sea ice. This detailed spatial resolution highlights its potential for monitoring of temperature patterns in Antarctica.

## Data Availability

Python 3.8 was used for conversion of the MODIS products from HDF files to raster and all data handling and processing was thereafter done in R version 4.0.0. All data processing and modelling procedures are available as R scripts on a public Github repository: https://github.com/evabendix/AntAir-ICE. Using this code it is possible to download new available MODIS LST and IST scenes and apply the model to continue the near-surface air temperature dataset.
